# Enhanced Cycling Stability of Al-Doped Li_1.20_Mn_0.52–*x*_Al_*x*_Ni_0.20_Co_0.08_O_2_ as a Cathode Material for Li-Ion Batteries by a Supercritical-CO_2_-Assisted Method

**DOI:** 10.1021/acsomega.4c05087

**Published:** 2024-11-15

**Authors:** Ali Yalçın, Mehmet Oğuz Güler, Muslum Demir, Mehmet Gönen, Mesut Akgün

**Affiliations:** † Department of Chemical Engineering, Faculty of Engineering and Natural Sciences, Süleyman Demirel University, Isparta 32260, Türkiye; ‡ Department of Metallurgical & Materials Engineering, Faculty of Engineering, Sakarya University, Sakarya 54197, Türkiye; § Natriontech Energy Technologies Inc., Technopark İstanbul, İstanbul, Pendik 34342, Türkiye; ∥ Department of Chemical Engineering, Faculty of Engineering, Bogazici University, İstanbul 34342, Türkiye; ⊥ TUBITAK Marmara Research Center, Material Institute, Gebze 41470, Türkiye; # Department of Chemical Engineering, Faculty of Chemical and Metallurgical, Yıldız Technical University, İstanbul 34210, Türkiye

## Abstract

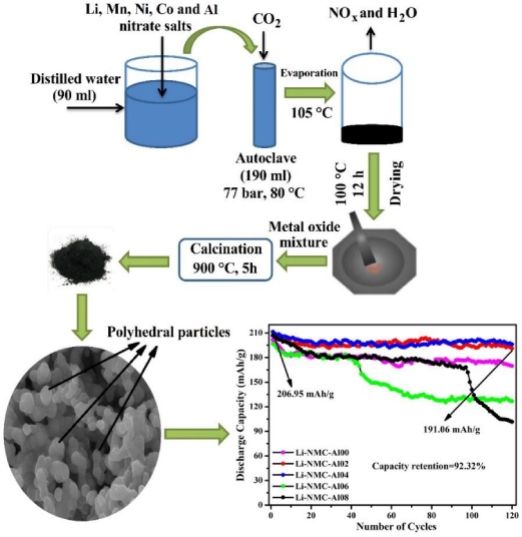

Lithium-rich layered oxide materials (Li-NMC) are considered a potential cathode material for next-generation batteries, thanks to their high theoretical specific capacity. Large potential drop and capacity loss after long cycles are the main obstacles to expanding commercial utilization of Li-NMC. In the past decade, great efforts have been made to overcome those issues of Li-NMCs. In this study, Al-doped Li_1.20_Mn_0.52–*x*_Al_*x*_Ni_0.20_Co_0.08_O_2_ cathode materials are for the first time synthesized by a supercritical-CO_2_-assisted method. Upon the electrochemical tests of Al-doped Li-rich NMCs, the optimal initial charge/discharge profile is obtained for the Li-NMC-Al02 cathode with 374.6/247.5 mAh/g compared with that of 320.7/235.1 mAh/g for the pristine Li-NMC-Al00 sample at the C/20 rate. In addition, the Li-NMC-Al02 cathode shows an enhanced rate-capability performance compared to the pristine sample at relatively low rates. When the current density is increased from C/10 to 3C, the charge/discharge capacity values of the Li-NMC-Al02 cathode are measured as 249.88/105.84 mAh/g. Last but not least, Li-NMC-Al02 demonstrates an excellent energy retention of 92.32%, which is notably higher than that of pristine Li-NMC-Al00 (86.4%) after 120 cycles at the C/20 rate. Overall, the present fabrication and doping strategy opens a new avenue for commercialization of Li-NMC cathode materials.

## 1 Introduction

Rechargeable lithium-ion batteries (LIBs) have attracted the attention of many researchers because they potentially have a large range of applications such as smart mobile phones, laptops, portable electronics, drones, unmanned aerial vehicles (UAVs), electric vehicles (EVs), and hybrid electric vehicles (HEVs).^1−4^ Especially, UAV, EV, and HEV technology require lithium-ion batteries that have high-energy density and can operate at high voltages.^5,6^ It needs to use new-generation lithium-ion batteries to meet the energy density required in these latest technologies. The Li-rich nickel manganese cobalt (NMC)-layered oxides are one of the next-generation cathode materials for LIBs due to their high-energy density, enhanced rate capability, high discharge capacity, and working at a high voltage up to 4.8 V.^7^ Li-rich NMC metal oxides are denoted as *x*Li_2_MnO_3_·(1 – *x*)LiMO_2_ (*M* = Mn, Ni, and Co, 0 < *x* < 1) formula, and they are of a reversible discharge capacity exceeding 250 mAh/g at a range of 2–4.8 V due to their high electrochemical properties.^8^ However, these Li-rich NMC oxides suffer from poor electrochemical properties, such as inferior cycling stability, poor rate capability, low discharge capacity, and voltage fading, thus hindering their use in next-generation technology applications.^9,10^ The Li-rich NMC consists of an arranged layered structure of Li_2_MnO_3_ (space group *C*2/*m*) and the octahedral sites of the close-packed oxygen arrays in LiMO_2_ (space group R3̅m).^11,12^ When charged above 4.5 V, Li^+^ in the structure of Li_2_MnO_3_ turns into Li_2_O at the end of the irreversible reaction. It causes the migration of transition metal ions to lithium hosts at the cathode, which leads to poor electrochemical properties and structural instability during cycling.^11^ In addition, the other shortcomings are side reactions between the electrode–electrolyte, HF attacks, the formation of the SEI or CEI layer, and dendritic growth.^5,13−15^ Eliminating these deficiencies is a requirement for the use of Li-rich NMC cathode materials in advanced technology.^7^ To solve those problems, various approaches have been suggested, such as metal coating,^16,17^ hybrid coating,^18^ carbon coating,^19^ heterostructure,^20^ cation doping,^7,11,21^ and core–shell structure,^22^ in the literature.

The bulk lattice cation doping is a simple and applicable method that transitional metals can homogeneously distribute into the crystal lattice of the cathode material.^7,13^ The scientific reasons behind cation doping into cathode materials are explained as follows:

i) The binding energy of doping metals to the oxygen element is higher than the binding energy of Mn, Ni, and Co in Li-rich NMC.^7,13,23^

ii) The atomic radius of the doping metals is higher than that of Mn, Ni, and Co.^13,24^

iii) Doping metals should not participate in redox reactions occurring at the cathode.^1^

The previous studies reported that Al doping is a beneficial method for stabilizing the structure of Li-rich NMC cathode materials and the cathode electrode/electrolyte interphase.^25^ Al is one of the metals that do not participate in redox reactions in the cathode electrode. In addition, the binding energy of Al–O is greater than that of the Mn–O, Ni–O, and Co–O binding energies. This strong binding between Al and oxygen sheets makes stability to the framework of Li-rich NMC and enhances the electrochemical properties of the Li-ion battery during cycling.^23,26^ Tang and Lang (2016) synthesized a series of Al-doped Li[Li_0.2_Ni_0.15_Mn_0.55_Co_0.1–*x*_Al_*x*_]O_2_ by replacing cobalt with aluminum in Li[Li_0.2_Ni_0.15_Mn_0.55_Co_0.1_]O_2_. It was reported that Al doping has significantly enhanced the cycle performance and rate performance of related cathode materials, but their initial discharge capacity decreases slightly. The initial discharge capacity of the 0.05 Al-doped cathode material was measured as 231.7 mAh/g at 25 °C and 275.8 mAh/g at 55 °C, respectively, both at 0.1 C and 2–4.8 V. Moreover, after 30 cycles, capacity retentions for the 0.05 Al-doped cathode material were determined to be 98% for 25 °C and 96% for 55 °C at 0.5 C within the potential range from 2.0 to 4.8 V. These results show that doping a suitable amount of Al into the cathode material enhances the structural stability of cathode materials.^26^ Li_1.16_Mn_0.54_Ni_0.13_Co_0.13_Al_0.04_O_2_, Li_1.2_Mn_0.50_Ni_0.13_Co_0.13_Al_0.04_O_2_, Li_1.2_Mn_0.54_Ni_0.09_Co_0.13_Al_0.04_O_2_, and Li_1.2_Mn_0.54_Ni_0.13_Co_0.09_Al_0.04_O_2_ materials were synthesized by replacing each element with Al in the Li_1.2_Mn_0.54_Ni_0.13_Co_0.13_O_2_ material. It was reported that Al doping does not significantly change the structure and morphology of the material but enhances the cycling performance by improving the structural stability of the material. The initial specific capacities of Li_1.16_Mn_0.54_Ni_0.13_Co_0.13_Al_0.04_O_2_ material were determined as 271.0 mAh/g for 0.1 C and 185 mAh/g for 0.5 C at 2–4.8 V. The cycle retention rate of Li_1.16_Mn_0.54_Ni_0.13_Co_0.13_Al_0.04_O_2_ was 97.3% after 100 cycles. As a result, it was found that 0.04 mol Al doping to substitute Li in the cathode material can enhance the cycle performance and stability of Li_1.2_Mn_0.54_Ni_0.13_Co_0.13_O_2_ material.^27^ In another study, the Al-doped Li[Ni_0.78_Co_0.1_Mn_0.1_Al_0.02_]O_2_ cathode material was synthesized by replacing Ni with 0.02 mol of Al in the Li[Ni_0.8_Co_0.1_Mn_0.1_]O_2_ cathode material with higher Ni content. The retention capacities of the bare NCM811 and the Al-doped NCM811 were determined as 87.32%–94.38% after the 50th cycle at 60 °C and 73.59%–96.15% after the 100th cycle at 20 °C, respectively. The enhanced cycling performance of Al-doped NCM811 was attributed to the improved thermal and structural properties of the cathode material.^28^

To date, various routes such as the wet chemical method, solid-state reaction method, coprecipitation, sol–gel technique, hydrothermal synthesis, Pechini method, and microwave-assisted method have been investigated for the Li-rich NMC synthesis.^5,29^ In our previous study, Li-rich NMC material was synthesized using a supercritical CO_2_-assisted synthesis technique, and the scientific reasons behind this method were explained.^9,30−32^ In another study, the denitration process has been applied to prepare different metal oxides with various metals.^33^ While nitric acid was used to decrease the pH in the liquid phase in the denitration process, CO_2_ in an aqueous phase was utilized in the supercritical CO_2_-assisted method, owing to its pH-lowering effect. CO_2_ dissolves by forming carbonic acid in the aqueous phase. This acid produces protons that increase the solubility and a fast dissolution rate of the reactants in addition to bicarbonate anions. Furthermore, this bicarbonate anion dissociates into carbonate ions and H^+^. The reaction mechanism representing the dissolution of CO_2_ in the aqueous phase is shown in eq 1–3.^30,31^ To the best of our knowledge, there is no study regarding the synthesis of an Al^3+^-doped Li-rich NMC via the supercritical CO_2_-assisted method. Herein, for the first time, Al^3+^ doping into the crystal lattice of Li_1.20_Mn_0.52–*x*_Al_*x*_Ni_0.20_Co_0.08_O_2_ was carried out by using the supercritical CO_2_-assisted method.
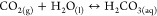
1
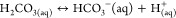
2
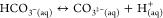
3

This study aims to synthesize a Li_1.2_Mn_0.52_Ni_0.20_Co_0.08_O_2_ cathode material using the supercritical CO_2_-assisted method. Al-doped Li_1.20_Mn_0.52–*x*_Al_*x*_Ni_0.20_Co_0.08_O_2_ (*x* = 0.00, 0.02, 0.04, 0.06, and 0.08) was also obtained by adding Al cations into the reaction medium. The Li_1.2_Mn_0.52_Ni_0.20_Co_0.08_O_2_ cathode material having a low cobalt rate was preferred due to the environmentally unfriendly, expensive, and limited natural cobalt sources. XRD, SEM/EDS, and ICP/OES analyses were performed to characterize the crystalline structure, morphology, and composition of the synthesized cathode. Electrochemical analyses and electrochemical properties of half-cells prepared from the synthesized cathode materials were determined. The substitution mechanism between Al^3+^ and Mn^4+^ ions in Li_1.20_Mn_0.52–*x*_Al_*x*_Ni_0.20_Co_0.08_O_2_ has also been explained.

## 2 Experimental Procedures

### 2.1 Synthesis of Cathode Materials

**Figure 1:**
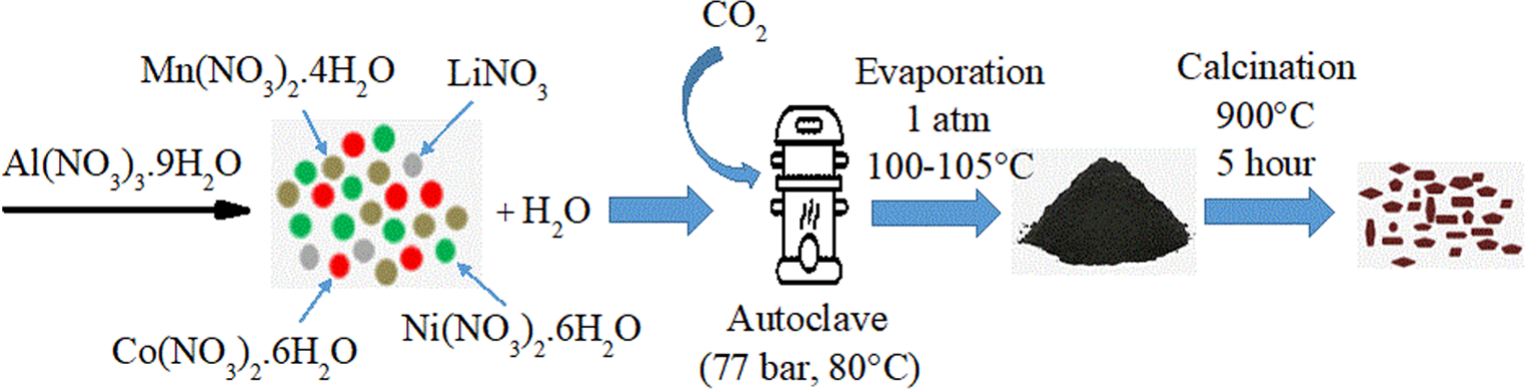
Experimental setup for a supercritical-CO_2_-assisted synthesis.

As seen in Figure 1, Al-doped Li_1.20_Mn_0.52–*x*_Al_*x*_Ni_0.20_Co_0.08_O_2_ (*x* = 0.00, 0.02, 0.04, 0.06, and 0.08) cathode materials were synthesized using a supercritical CO_2_-assisted method in the pretreatment followed by thermal treatment. Stoichiometric amounts of Mn(NO_3_)_2_·4H_2_O (Merck, 98.5 wt %), Co(NO_3_)_2_·6H_2_O (Kimetsan, 99.1 wt %), Ni(NO_3_)_2_·6H_2_O (Kimetsan, 19.7% of nickel assay in mass), LiNO_3_ (Merck, +98 wt %), and Al(NO_3_)_3_·9H_2_O (Across Organics, 99+%) were weighed to synthesize pristine and Al-doped Li_1.20_Mn_0.52–*x*_Al_*x*_Ni_0.20_Co_0.08_O_2_ (*x* = 0.02, 0.04, 0.06, and 0.08) materials. All materials were dissolved in distilled water (90 mL) and then transferred into the reactor.

A stainless-steel reactor (190 mL) was settled in a water tub on a magnetic stirrer with a heating and mixing control system (Wise Stir MSH-20D). To reach 77 bar, the required amount of CO_2_ (dry ice) was added to the reactor. The CO_2_ amount was calculated using the Peng–Robinson equation of state.^34^ The stirring rate (300 rpm) and temperature (80 °C) were fixed using a controller. In those process conditions, the mixture was exposed to pretreatment in the presence of CO_2_.

After pretreatment for 2 h, a solution with low pH was evaporated at an elevated temperature (100–105 °C). Cation metals in the solution were crystallized by evaporation, which formed a metal oxide mixture. Then, the crystallized powders were calcined in an oven for 5 h at 900 °C in an air atmosphere.^9,30,32,34^ The final products were stored in a desiccator for characterization and electrochemical tests.^30^ The final products were denoted as Li-NMC-Al02, Li-NMC-Al04, Li-NMC-Al06, and Li-NMC-Al08, where Li, N, M, C, and Al cite to Li, Ni, Mn, Co, and Al elements and numbers refer to mole amounts of Al (0.02, 0.04, 0.06, and 0.08), respectively. The pristine sample was named as Li_1.2_Mn_0.52_Ni_0.20_Co_0.08_O_2_ (Li-NMC-Al00). Figure 1 depicts the experimental setup used for all of the runs.

### 2.2 Characterization of Samples

To study the crystalline structure and phase purity of all final products, XRD equipment (Bruker D8 Advance Twin–Twin) equipped with CuKα recorded XRD spectra diffraction data in the 2θ range of 10–80°. Scanning electron microscopy (SEM, Zeiss Sigma 300) equipped with energy-dispersive X-ray spectroscopy (EDS) utility was employed to confirm surface morphologies such as particle shape, size, and average particle size. ICP-OES (PerkinElmer Optima 2100 DV) was used to detect the molar composition of Mn, Al, Ni, and Co elements in the structure of Li_1.20_Mn_0.52–*x*_Al_*x*_Ni_0.20_Co_0.08_O_2_.

### 2.3 Electrochemical Test Studies of Half-Cell Batteries

Half-cells (CR2032) were utilized for conducting electrochemical tests of all LIBs prepared from synthesized cathode materials. The slurry was prepared with active Li_1.20_Mn_0.52–*x*_Al_*x*_Ni_0.20_Co_0.08_O_2_ (*x* = 0.00, 0.02, 0.04, 0.06, and 0.08) materials, carbon black, and PVDF binder (polyvinylidene tetrafluoroethylene) in wt % of 83:10:7, with *N*-methyl-2-pyrrolidone solvent (NMP). It was coated onto Al foil (18 μm thickness) as a current collector using a coating device and subsequently dried at 80 °C for 18 h in a vacuum oven. The coated Al foil positive electrode was punched into circular discs (19 mm). Half-cells were fabricated with the cathode electrode, a porous polypropylene separator (Celgard 2400), an electrolyte (1 M LiPF_6_ dissolved in v/v = 50:50 solution of EC (ethylene carbonate), DC (diethyl carbonate)), and a lithium metal anode in an argon-filled glovebox (O_2_ and H_2_O < 1 ppm). All electrochemical measurements of half-cells were conducted at room temperature.

A Neware BTS4000 test system was used for determining the galvanostatic charge–discharge tests of all half-cells. Measurements were carried out in the range of 2.0–4.8 V (vs Li/Li^+^) at C/20–3 C current densities (1 C = 240 mA/g). EIS measurements were determined using Gamry Instruments. EIS tests were carried out at frequencies in the range of 10 kHz to 10 MHz with a perturbation amplitude of 10 mV. CH Instruments Inc. was also used for the cyclic voltammetry (CV) tests, performed at 2.0–4.8 V and a scanning rate of 0.1 mV/s. The cycling algorithm reported in previous studies was used for determining the charge/discharge of half-cells.^9,30^

## 3 Results and Discussion

### 3.1 Physical Characterization

**Figure 2:**
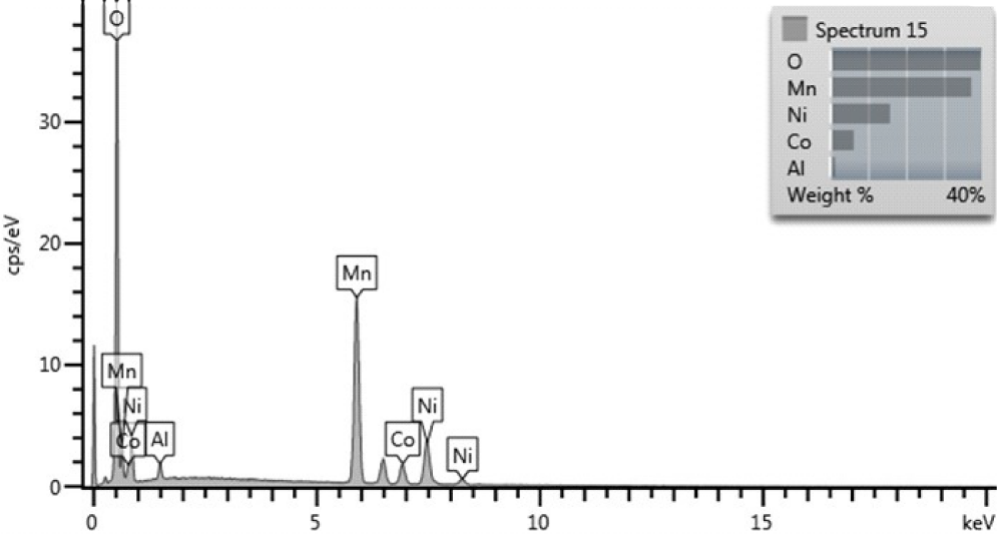
EDS spectrum of the Li-NMC-Al02 sample.

The elemental composition values in the structures of Li-NMC-Al00, Li-NMC-Al02, and Li-NMC-Al04 samples are given in Table 1. Since the atomic mass of the Li element is low, the presence of this element has not been detected by EDS (Figure 2). Therefore, taking Li as the reference element, the composition values of other elements were calculated from the data obtained by EDS analysis. From the data obtained by ICP-OES analysis, it can be seen that the chemical composition (molar composition) of Li-NMC-Al00 and Li-NMC-Al04 samples is Li_1.020_Mn_0.520_Al_0.000_Ni_0.200_Co_0.080_ and Li_1.090_Mn_0.462_Al_0.037_Ni_0.208_Co_0.082_, respectively. As seen from Table 1, the molar composition of the Mn, Al, Ni, and Co elements in the structure is close to the theoretical composition values. However, due to the evaporation of Li at temperatures higher than 900 °C,^30^ the molar composition value in the structure appears to be slightly low in the ICP-OES analysis. In the literature, the chemical composition of Al-doped Li_1.01_(Ni_0.39_Mn_0.40_Co_0.15_Al_0.06_)_0.99_O_2_,^1^ Li_0.95_Ni_0.92_Co_0.06_Al_0.02_O_2−δ_,^35^ LiNi_0.79_Co_0.15_Mn_0.05_Al_0.01_O_2_,^36^ and Li_1.25_Mn_0.548_Ni_0.132_Co_0.024_Al_0.031_O_2_^37^ cathode materials has been determined based on the data obtained from ICP analysis. According to the results of EDS and ICP-OES, it can be said that the calculated chemical composition of the Li_1.080_Mn_0.505_Al_0.037_Ni_0.210_Co_0.081_ cathode material synthesized at 900 °C and 5 h in this study is close to the theoretical composition compared with the samples synthesized in the literature.

**Table 1:** Composition of Elements in the Li_1.20_Mn_0.52–*x*_Al_*x*_Ni_0.20_Co_0.08_O_2_ Samples

**Analysis**	**Al content**	**Li**	**Mn**	**Al**	**Ni**	**Co**
Theoretical		1.200	0.52 – *x*	*x*	0.200	0.080
EDS	0.00 Al	-	0.520	0.000	0.188	0.071
EDS	0.02 Al	-	0.502	0.024	0.202	0.083
EDS	0.04 Al	-	0.485	0.038	0.208	0.085
ICP-OES	0.00 Al	1.020	0.520	0.000	0.200	0.080
ICP-OES	0.04 Al	1.090	0.462	0.037	0.208	0.082

**Figure 3:**
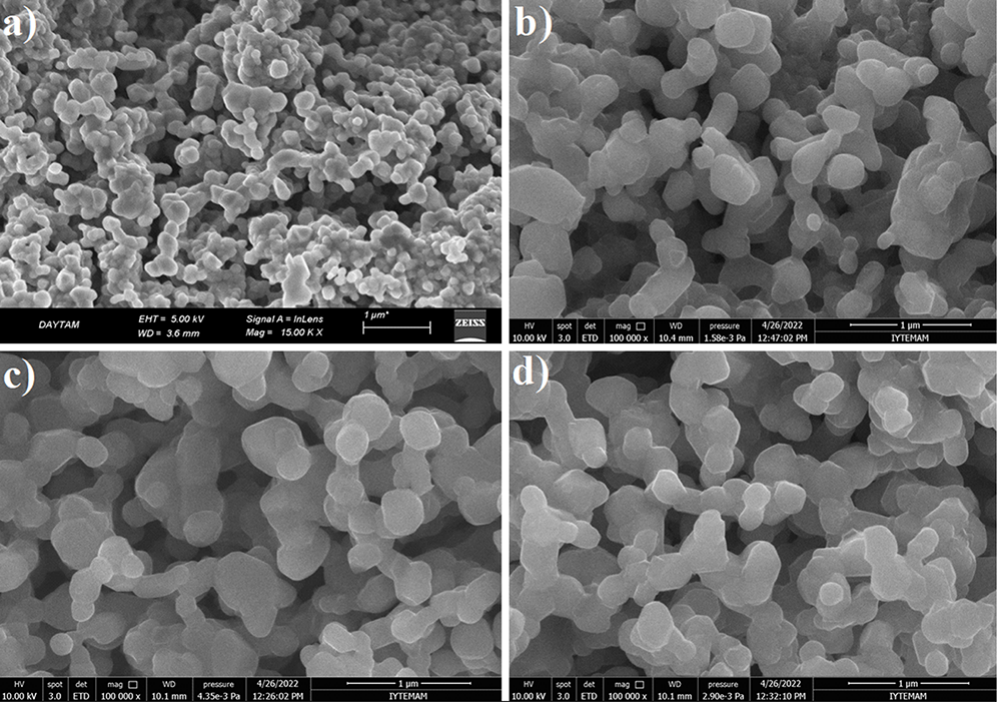
SEM images of (a) Li-NMC-Al00, (b) Li-NMC-Al02, (c) Li-NMC-Al04, and (d) Li-NMC-Al06 cathode materials.

SEM images of Li_1.20_Mn_0.52–x_Al_*x*_Ni_0.20_Co_0.08_O_2_ (x = 0–0.06) cathode materials are shown in Figure 3. It can be seen from the SEM images in Figure 3 that the surface morphologies of the four samples, such as particle size and distribution, are similar to each other. The average size of the particles varies between 100 and 300 nm. For these four samples, the calcination temperature and time (900 °C and 5 h) are the same, and the only feature that changes is the increase in the amount of Al doping in the structure. Even if the amount of Al doping increases, the morphologies of the particles are similar to each other, and there is no change in the particle size of all samples. It can be predicted that the effect on the electrochemical properties of these samples due to their surface morphology would be similar. However, since the most important parameter affecting electrochemical performance is the doping amount, it would be meaningless to predict the effect of surface morphology here. In a study by Iftekhar et al., the particle sizes of Li_1.16_Ni_0.167_Mn_0.50–*x*_Al_*x*_Co_0.167_O_2_ (*x* = 0, 0.01, and 0.02) cathode materials vary between 0.1 and 0.5 μm and consist of irregular particles.^38^ They reported that given the similarities in morphologies between these particles, effects on electrochemical performance cannot be reconciled with particle size. In another study,^39^ it was determined that the morphologies of pristine and Al-doped Li_1.2_Mn_0.56_Ni_0.16_Co_0.08–*x*_Al_*x*_O_2_ cathode materials were similar to each other and uniform particle distribution. The particle size of all samples is approximately 200–300 nm, and no change in morphology was observed with Al doping. In Tang and Zhang’s study,^26^ it was determined that the synthesized Li[Li_0.2_Ni_0.15_Mn_0.55_Co_0.1–*x*_Al_*x*_]O_2_ (*x* = 0, 0.025, 0.050, and 0.075) cathode materials had a smooth surface, similar morphology, uniform particle size, and distribution. In Li-rich NMC cathode materials, the small particle size means that the material has a larger specific surface area and creates a larger contact area with the electrolyte. Thus, more metal ions can be introduced into the electrochemical reaction to effectively reduce electrode polarization. This results in improved electrochemical properties, resulting in good rate performance and cycle performance.^26^ In this study, the surface morphologies of the Al-doped Li_1.2_Mn_0.52–*x*_Al_*x*_Ni_0.2_Co_0.08_O_2_ (*x* = 0.00, 0.02, 0.04, 0.06, and 0.08) samples synthesized under 900 °C and 5 h calcination conditions are similar to the morphologies encountered in the literature.

**Figure 4:**
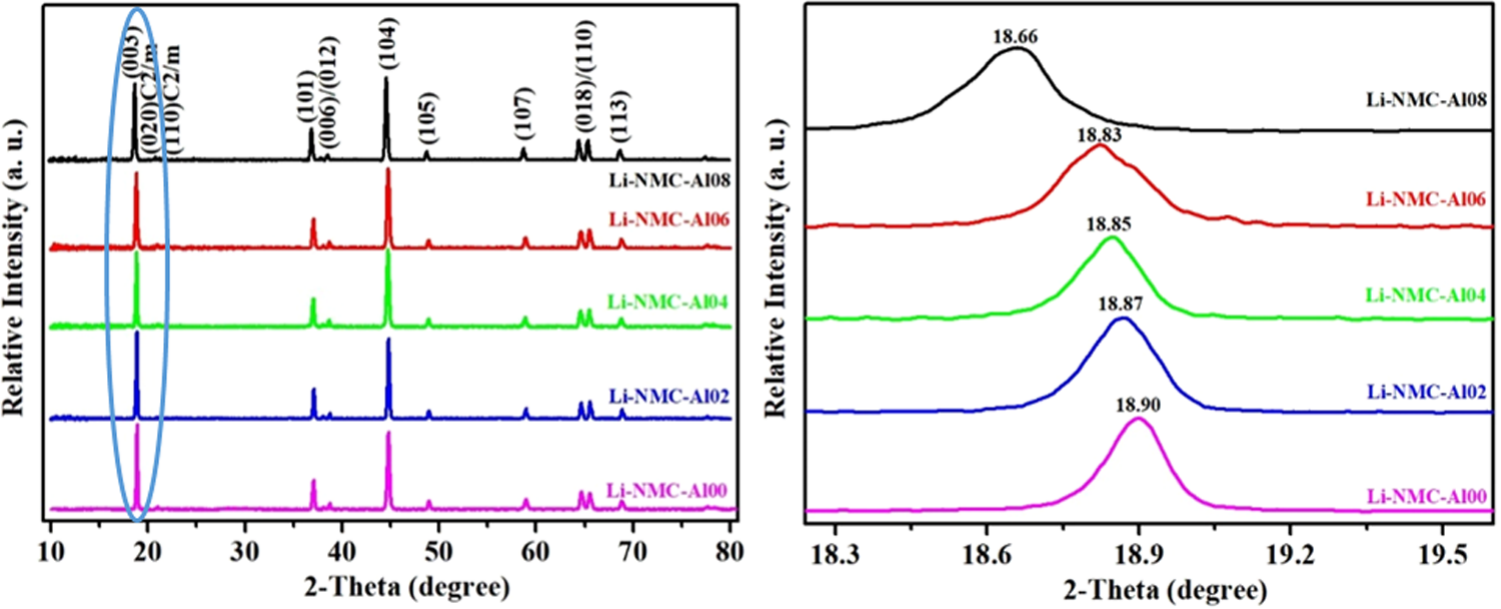
XRD patterns of Li_1.20_Mn_0.52–*x*_Al_*x*_Ni_0.20_Co_0.08_O_2_ (*x* = 0–0.08) cathode materials.

The XRD patterns of Al-doped Li_1.2_Mn_0.52–*x*_Al_*x*_Ni_0.2_Co_0.08_O_2_ (*x* = 0.00, 0.02, 0.04, 0.06, and 0.08) samples are shown in Figure 4, and all XRD strong diffraction peaks of the samples are identified as hexagonal α-NaFeO_2_ structure (space group: R3̅m, No. 166). It is understood from the XRD patterns that a small amount of Al was successfully replaced by Mn. In Figure 4, the XRD pattern of pristine Li-NMC-Al00 without Al doping is the same as the peaks of other doped products and is also similar to the diffraction peaks of the hexagonal α-NaFeO_2_ structure. Additionally, no impurity peaks are observed in the XRD patterns of all samples with or without Al doping. Characteristic main peaks^40^ of Al_2_O_3_ were not observed in the doped samples at 35.45° and 57.79°. In the Li_1.2_Mn_0.52–*x*_Al_*x*_Ni_0.2_Co_0.08_O_2_ structure, the doping of Al into the lattice results in expansion of the *c*-axis. As the Al content increases, a gradual leftward shift to lower angles is observed at the (003) peak of each sample,^41^ and this means that Al successfully joined the hexagonal structure of cathode material. The single most obvious difference between the samples is the degree of cation mixing of the I(003)/I(104) ratio. The I(003)/I(104) peak intensity ratios of the products according to the Al doping mole amounts (0.00, 0.02, 0.04, 0.06, and 0.08) are 1.11, 1.09, 0.98, 0.96, and 0.93, respectively. Since the degree of cation mixing affects the electrochemical performance in a long cycling life, the effect of the doping amount was evaluated based on the electrochemical test results.

In the literature, Al-doped Li[Li_0.2_Ni_0.15_Mn_0.55_Co_0.1–*x*_Al_*x*_]O_2_ samples were synthesized by the sol–gel method.^26^ It has been determined that these cathode materials produced at low doping rates are similar to the hexagonal α-NaFeO_2_ structures of Li-rich NMC cathode materials in the literature. Since the ionic diameter of Al is smaller than the diameter of Sn, doping Al to the hexagonal crystal lattice is easier than Sn, and no extra peaks of Al_2_O_3_ were found in the XRD patterns of Al-doped Li-NMC cathode materials in the literature.^26,27,38,42^ There is no impurity in the XRD diffraction peaks of the Al-doped Li_1.2_Mn_0.52–*x*_Al_*x*_Ni_0.2_Co_0.08_O_2_ (*x* = 0.00, 0.02, 0.04, 0.06, and 0.08) samples synthesized at 900 °C and 5 h in this study, and when compared with the studies in the literature, it can be concluded that Al is included in the hexagonal structure.

### 3.2 Electrochemical Tests

**Figure 5:**
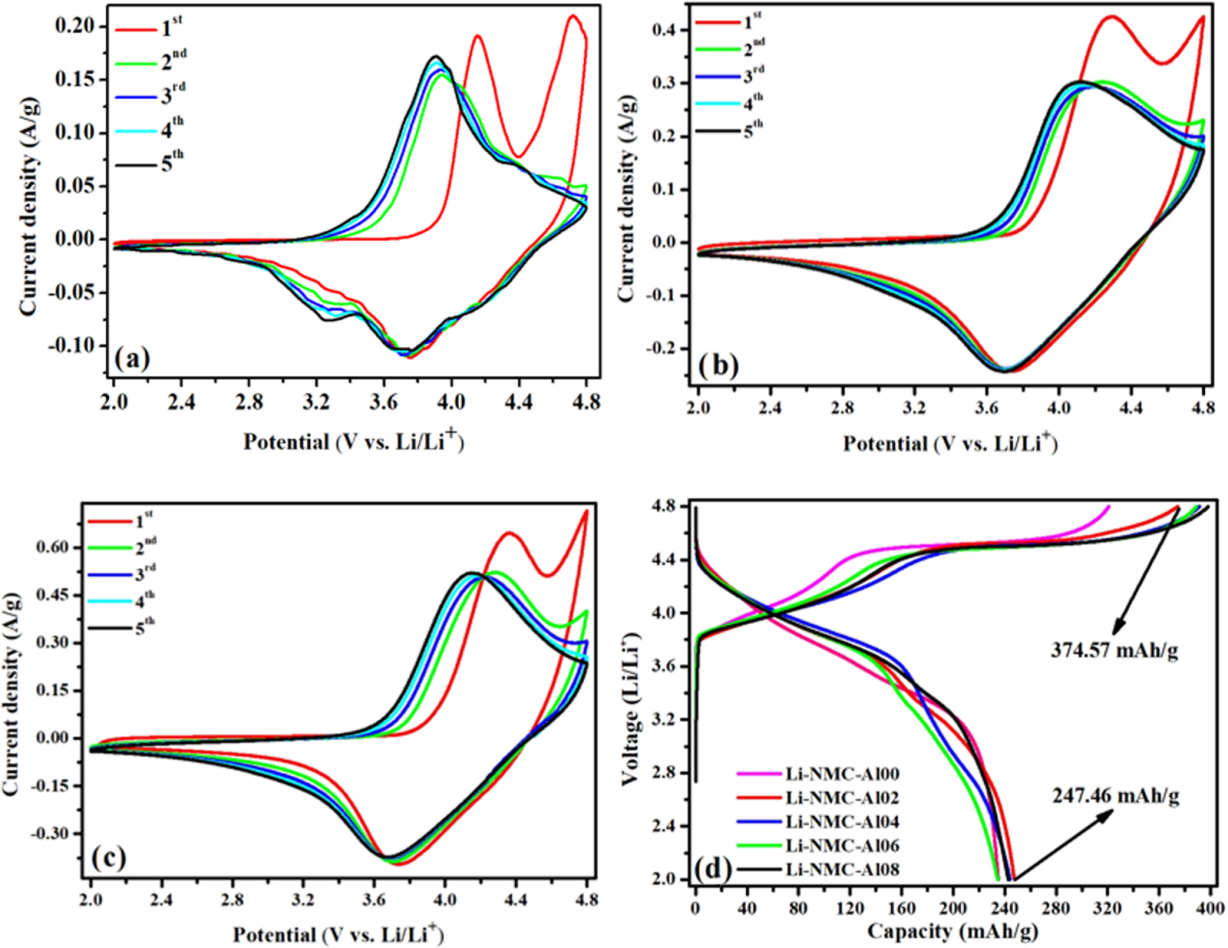
CV curves of Li_1.20_Mn_0.52–*x*_Al_*x*_Ni_0.20_Co_0.08_O_2_ samples (a) Al00, (b) Al02, and (c) Al04 for 5 cycles and (d) initial charge/discharge curves of all samples at C/20.

Cyclic voltammetry analyses (CV) of pristine Li-NMC-Al00, Li-NMC-Al02, and Li-NMC-Al04 cathode materials are shown in Figures 5a–c. These samples exhibited two oxidation peaks between 4.0 and 4.8 V at the first charge. These peaks represent oxidation reactions of Co^3+^ and Ni^2+^ and irreversible deactivation of the Li_2_MnO_3_ component.^8,11^ The two reduction peaks at around 3.74 V correspond to the reduction of Ni^3+^ and Co^4+^ cations (Figure 5a). The broad peak at 3.25 V is formed by the reduction of the Mn^4+^ cation. The charge loss resulting from irreversible oxygen loss during the first charging process is compensated here (Figure 5a).^8,27^ Due to irreversible activation of Li_2_MnO_3_, the second peak that occurs at ∼4.72 V in the first charge vanishes in the second charge (Figures 5a–c). The reaction mechanisms (eqs 4–6) occurring in the structure of Li-NMC-Al00 and Li-NMC-Al02 during the first cycle were shown as follows:^43^
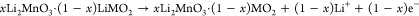
4
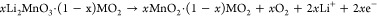
5
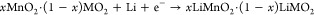
6

In Li-rich NMC cathode materials, voltage loss is generally observed after high cycling. Due to the poor cycle stability of Li_1.2_Mn_0.52_Ni_0.20_Co_0.08_O_2_^8^ and the transformation from layered to spinel phase,^11^ fluctuations are observed in the overlap of CV curves after the first cycle. As seen in Figures 5a–c, the cycle stability of the pristine Li-NMC-Al00 cathode material was increased by doping 0.02 and 0.04 Al. It is seen that the cyclic voltammetry curves of Li-NMC-Al02 and Li-NMC-Al04 samples overlap more regularly for the first 5 cycles.

The first charge/discharge capacity values of Li_1.20_Mn_0.52–*x*_Al_*x*_Ni_0.20_Co_0.08_O_2_ (*x* = 0–0.08) cathode materials at C/20 are shown in Figure 5d. For these materials, the initial charge capacity values were measured as 320.66, 374.57, 391.36, 388.77, and 398.07 mAh/g, respectively, at C/20 current density, while at the same current density, the initial discharge values were measured as 235.06, 247.46, 244.13, 234.51, and 242.76 mAh/g. Among the materials, Li-NMC-Al02 and Li-NMC-Al04 showed the best initial charge/discharge profiles. It can be seen in Figure 5d that as the doping amount increases, the initial charge/discharge values generally raise. During the charge–discharge process, Al^3+^ ions act as positive charge centers, stabilizing the matrix structure and significantly inhibiting phase transitions. The doping of Al^3+^ into the structure enhances the reversibility of the H2–H3 phase transition, mitigating the structural collapse caused by uneven stresses. Consequently, the material maintains a wide lithium layer spacing, leading to higher capacity.^41^ In the literature, the initial charge/discharge values of the LiNi_0.79_Co_0.15_Mn_0.05_Al_0.01_O_2_ cathode material at 0.1 C were found to be 217.6 and 208.1 mAh/g, respectively.^36^ For the Li[Li_0.2_Ni_0.15_Mn_0.55_Co_0.05_Al_0.05_]O_2_ sample, these values are 328.1 and 231.7 mAh/g at 0.1 C.^26^ It was reported that the initial charge/discharge values of the Li_1.16_Mn_0.54_Ni_0.13_Co_0.13_Al_0.04_O_2_ sample at 0.1 C current rate were 372.3 and 271.0 mAh/g, respectively.^27^

**Figure 6:**
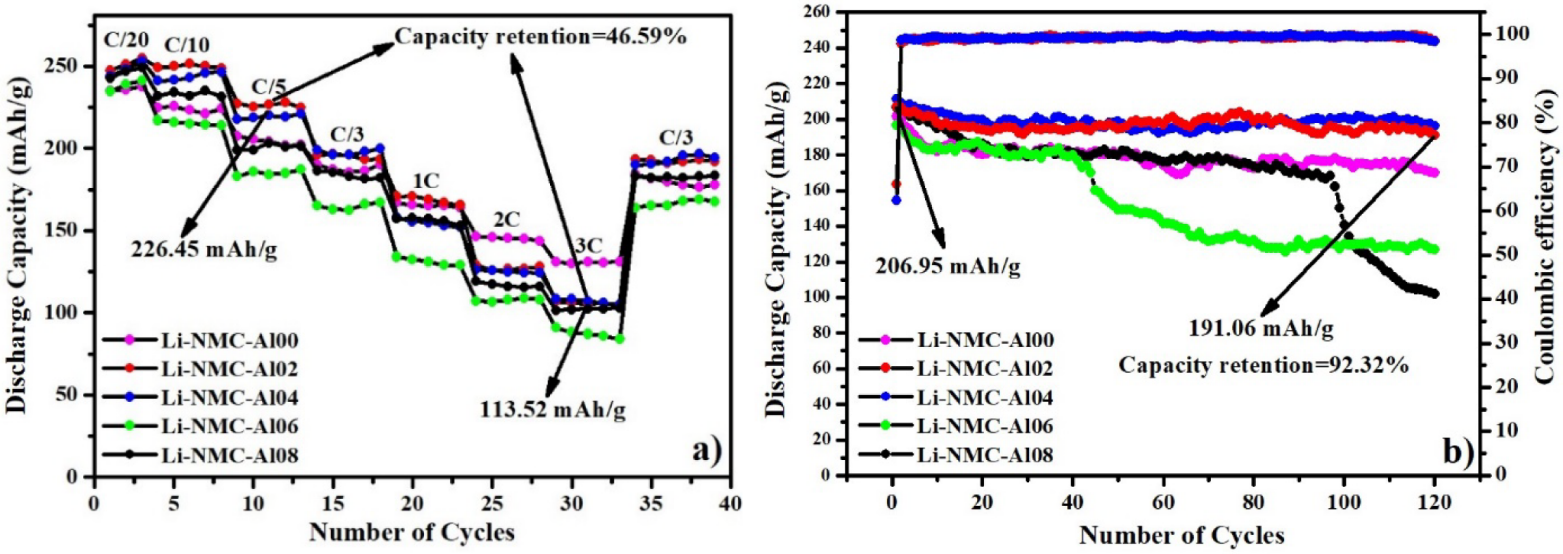
(a) Rate capabilities (C/20–3 C) and (b) cycle performances/Coulombic efficiency at C/3 of Li_1.20_Mn_0.52–*x*_Al_*x*_Ni_0.20_Co_0.08_O_2_ (*x* = 0–0.08) samples.

In Figure 6a, the rate performance of Li_1.20_Mn_0.52–*x*_Al_*x*_Ni_0.20_Co_0.08_O_2_ (*x* = 0–0.08) cathode materials at different C/20–3 C current densities is shown. The capacity values of the Li-NMC-Al00 and Li-NMC-Al02 were decreased from 201.5 to 130.8 and 226.45 to 113.52 mAh/g, respectively, when the current density was increased from C/5 to 3 C. However, when the current density is returned to the C/3 rate after 35 cycles, the Li-NMC-Al02 cathode material provides a significantly higher discharge capacity of 195.2 mAh/g, compared to those (176 mAh/g) of pristine Li-NMC-Al00. According to the rate capacity results increased from C/5 to 3 C, the capacity retention of the Li-NMC-Al02 cathode material was calculated as 46.59%.

The cycling stability of Li_1.20_Mn_0.52–*x*_Al_*x*_Ni_0.20_Co_0.08_O_2_ (*x* = 0–0.08) samples at C/3 is shown in Figure 6b. After 120 cycles, the capacity retention performances are 86.37%, 92.32%, 92.79%, 64.60%, and 49.21%, respectively. The best electrochemical performance was exhibited by Li-NMC-Al02 and Li-NMC-Al04 cathode materials, and the Coulombic efficiencies of both materials were 66.07–62.4% for the first cycle and 98.50–98.31% for the 120th cycle, respectively (Figure 6b). Li-NMC-Al06 and Li-NMC-Al08 materials experienced significant discharge capacity fading as the cycle increased. Excessive amounts of Al within the Li_1.20_Mn_0.52_Ni_0.20_Co_0.08_O_2_ lattice can increase the activation energy for Li^+^ movement, further restricting intercalation/deintercalation behavior. Additionally, the presence of excess Al leads to an increase in Mn^3+^, which subsequently exacerbates its dissolution and results in the degradation of cycling performance.^44^ It was determined that these materials exhibited the best initial charge–discharge, rate performance, and cycle performance/Coulombic efficiency at the C/3 rate. A comparison of performance tests of samples synthesized in this study and the literature is exhibited in Table 2. As seen in Table 2, it can be said that Li_1.20_Mn_0.50_Al_0.02_Ni_0.20_Co_0.08_O_2_ and Li_1.20_Mn_0.48_Al_0.04_Ni_0.20_Co_0.08_O_2_ synthesized in this study have excellent performances when evaluated in terms of both initial discharge capacity and capacity retention.

**Table 2:** Comparison of Electrochemical Tests for Al-doped Cathode Materials

Materials	Morphology of samples	Current density	IDC (mAh/g)	Capacity retention	Reference
Li_1.2_Mn_0.50_Al_0.02_Ni_0.2_Co_0.08_O_2_	100–300 nm	C/20	247.5	92.32% after 120 cycles	This study
		C/10	249.9		
Li_1.2_Mn_0.48_Al_0.04_Ni_0.2_Co_0.08_O_2_	100–300 nm	C/20	245.0	92.79% after 120 cycles	This study
		C/10	247.2		
Li[Li_0.2_Ni_0.15_Mn_0.55_Co_0.05_Al_0.05_]O_2_	200–400 nm	0.1 C	231.7	98% after 30 cycles	26
1% Al-doped LiNi_0.76_Mn_0.14_Co_0.10_O_2_	-	1 C	210.0	89.8% after 400 cycles	25
Li_1.23_Mn_0.54_Ni_0.13_Co_0.07_Al_0.03_O_2_	200–400 nm	C/10	∼235.0	∼95.7% after 200 cycles	37
Li(Li_0.2_Ni_0.13_Co_0.13_Mn_0.52_Al_0.02_)O_2_	-	0.2 C	225.8	85.5% after 100 cycles	45
Li_1.16_Mn_0.54_Ni_0.13_Co_0.13_Al_0.04_O_2_	150–200 nm	0.1 C	271.2	97.3% after 100 cycles	27
Li_1.2_Mn_0.56_Ni_0.16_Co_0.03_Al_0.05_O_2_	200–300 nm	0.2 C	225.4	99.3% after 100 cycles	(39)
Li[Ni_0.78_Co_0.1_Mn_0.1_Al_0.02_]O_2_	6.38 μm	1 C	171.7	96.15% after 100 cycles	(28)
LiNi_0.92_Co_0.06_Al_0.02_O_2_	12 μm	C/10	172.0	90% after 100 cycles	(35)
Li[Ni_0.75_Co_0.09_Mn_0.14_Al_0.02_]O_2_	∼250 nm	0.1 C	203.0	95% after 100 cycles	(46)
IDC: initial discharge capacity					

**Figure 7:**
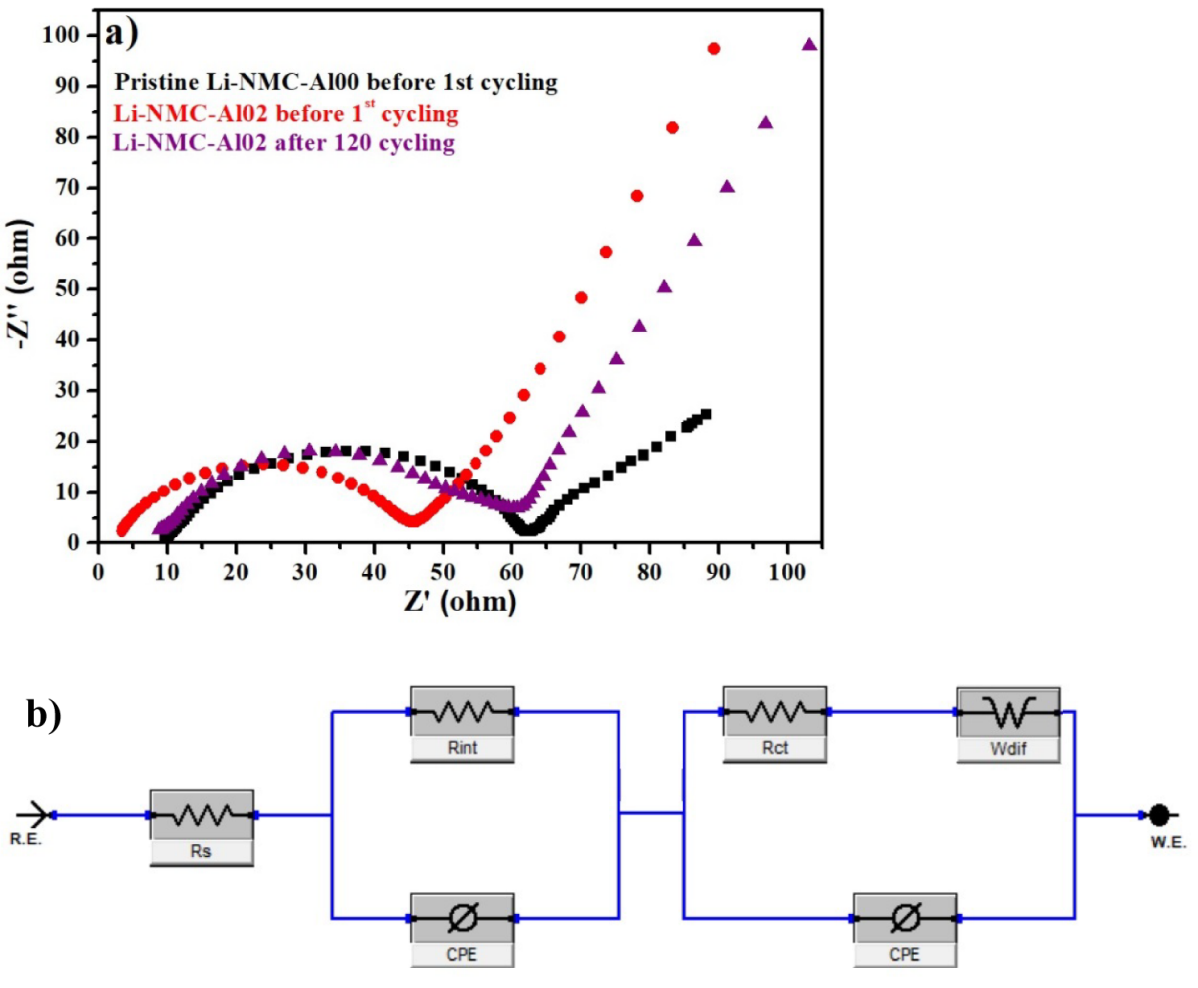
(a) Impedance spectra of pristine and Al-doped Li-NMC-Al02 at uncycle and at the end of the 120th cycle and (b) the equivalent circuit of half-cell obtained from the impedance spectra.

In addition to the equivalent circuit, the impedance measurement curves of Li_1.2_Mn_0.52_Ni_0.2_Co_0.08_O_2_ and Li_1.2_Mn_0.50_Al_0.02_Ni_0.2_Co_0.08_O_2_ cathode materials are shown in Figure 7. It shows the impedance curves of battery cells produced with Li-NMC-Al00 and Li-NMC-Al02 materials, which have never been charged/discharged, as well as cells that have been charged/discharged 120 times. In the figure, the semicircle, the inclined line, and the intersection between the semicircle and the real axis represent the charge transfer resistance (*R*_ct_) at the solid electrode/electrolyte interface, the Warburg impedance (*W*_o_), which represents the diffusion of Li^+^ in the layered oxide structure, and the solution resistance (*R*_s_), respectively.^7^

While the *R*_s_ value of the Li-NMC-Al02 material that has never been charged/discharged was 3.44, this value increased to 8.82 after 120 cycles (Table 3 and Figure 7a). For the same material, the *R*_ct_ value increased from 45.65 to 60.21 after 120 cycles. These values for the Li-NMC-Al04 material that has never been charged/discharged were 10.52 and 10.72 after 120 cycles. The *R*_ct_ value increases from 39.10 to 44.80 after 120 cycles. The *R*_ct_ value for the undoped Li-NMC-Al00 material is 63.80, and this value decreased with Al doping. This signifies that the half-cell produced with Li-NMC-Al02 and Li-NMC-Al04 materials is exposed to less resistance during 120 cycles, which facilitated fast the diffusion of Li^+^ between electrodes and electrolytes. These impedance measurements obtained are also consistent with the electrochemical test results. As seen in Table 4, an increase in the charge transfer resistance of Li_1.2_Mn_0.50_Al_0.02_Ni_0.2_Co_0.08_O_2_ and Li_1.2_Mn_0.48_Al_0.04_Ni_0.2_Co_0.08_O_2_ materials after 120 cycles is highly lower than that of Li-NMC samples reported in the literature.

**Table 3:** *R*_s_ and *R*_ct_ Values of the Li_1.20_Mn_0.52–*x*_Al_*x*_Ni_0.20_Co_0.08_O_2_ Materials

	***R*_s_ (Ω)**		***R*_ct_ (Ω)**	
**Cathodematerial**	**0. Cycle**	**120. Cycles**	**0. Cycle**	**120. Cycles**
Li-NMC-Al00	9.67	-	63.80	-
Li-NMC-Al02	3.44	8.82	45.65	60.21

**Table 4:** Comparison of *R*_ct_ Values of Al-doped Li-NMC Materials

**Samples**	**Cycle Number**	***R*_ct_ (Ω)**	**References**
Li_1.2_Mn_0.50_Al_0.02_Ni_0.2_Co_0.08_O_2_	uncycled	45.65	This study
	120th	60.21	
Li_1.2_Mn_0.48_Al_0.04_Ni_0.2_Co_0.08_O_2_	uncycled	39.1	This study
	120th	44.8	
Li(Li_0.18_Al_0.02_Ni_0.13_Co_0.13_Mn_0.54_)O_2_	100th	75.06	(45)
Li(Li_0.2_Ni_0.11_Al_0.02_Co_0.13_Mn_0.54_)O_2_	100th	218.8	
Li(Li_0.2_Ni_0.13_Co_0.11_Al_0.02_Mn_0.54_)O_2_	100th	195.1	
Li(Li_0.2_Ni_0.13_Co_0.13_Mn_0.52_Al_0.02_)O_2_	100th	107.2	
Li_1.14_(Ni_0.136_Co_0.136_Mn_0.544_)O_2_	1th	∼30.0	(42)
	50th	∼295.0	
Li_1.14_(Ni_0.136_Co_0.10_Al_0.03_Mn_0.544_)O_2_	1th	∼75.0	
	50th	∼302.0	
LiNi_0.76_Mn_0.14_Co_0.10_O_2_	10th	32.08	(25)
	50th	235.5	
1%Al doped-LiNi_0.76_Mn_0.14_Co_0.10_O_2_	10th	48.32	
	50th	65.26	
Li_1.16_Mn_0.54_Ni_0.13_Co_0.13_Al_0.04_O_2_	100th	79.5	(27)
Li_1.2_Mn_0.50_Ni_0.13_Co_0.13_Al_0.04_O_2_		344.6	
Li_1.2_Mn_0.54_Ni_0.09_Co_0.13_Al_0.04_O_2_		289.8	
Li_1.2_Mn_0.54_Ni_0.13_Co_0.09_Al_0.04_O_2_		259.3	
Li(Ni_0.84_Co_0.16_)_0.96_Al_0.04_O_2_	1th	24.8	(47)
	100th	122.6	

## 4 Conclusion

The intermediate products obtained by the supercritical CO_2_-assisted method were sintered at 900 °C and 5 h of calcination time, and Al-doped Li_1.2_Mn_0.52–*x*_Al_*x*_Ni_0.2_Co_0.08_O_2_ cathode materials were synthesized. The Al doping process was carried out in the reaction medium. Theoretical composition (Li_1.20_Mn_0.52–*x*_Al_*x*_Ni_0.20_Co_0.08_O_2_) of cathode materials (Li-NMC-Al02 and Li-NMC-Al04) is very close to the ones obtained from EDS and ICP/OES analysis. The initial charge/discharge capacity of cathode materials (Li-NMC-Al02 and Li-NMC-Al04) was measured as 374.57/247.46 and 391.36/244.13 mAh/g at C/20, respectively, which were better than those of other Al-doped samples. When the current density was increased from C/10 to 3 C, their charge/discharge capacity values were determined as 249.88/105.84 and 243.60/106.95 mAh/g, and the capacity retention values were calculated as 42.36% and 43.90%, respectively. The capacity retention values at C/3 for 120 cycles were calculated as 92.32% and 92.79%, respectively. At the end of 120 cycles, it was concluded that the cyclic voltammetry measurements of Li-NMC-Al02 and Li-NMC-Al04 cathode materials are compatible with the voltage values at which redox reactions occur in the literature. For the first 5 cycles, the CV curves of the cathode materials were found to overlap more regularly than those of the undoped Li-NMC-Al00.
